# Diversity and Distribution of Forest Ants (Hymenoptera: Formicidae) in Nepal: Implications for Sustainable Forest Management

**DOI:** 10.3390/insects12121128

**Published:** 2021-12-17

**Authors:** Indra Prasad Subedi, Prem Bahadur Budha, Ripu Mardhan Kunwar, Shambhu Charmakar, Sunita Ulak, Dhirendra Kumar Pradhan, Yam Prasad Pokharel, Sajeev Thavalathadathil Velayudhan, Shiroma Sathyapala, Illias Animon

**Affiliations:** 1 Central Department of Zoology, Institute of Science and Technology, Tribhuvan University, Kirtipur 44600, Nepal; indra.subedi@cdz.tu.edu.np; 2 Food and Agriculture Organization of the United Nations-Nepal, Pulchowk, Lalitpur 44700, Nepal; 3 Tropical and International Forestry, Technical University Dresden, Cotta Bau Pienner Str. 7, 01737 Tharandt, Germany; shambhuckr11@gmail.com; 4 Forest Research and Training Centre, Ministry of Forests and Environment, Kathmandu 44600, Nepal; sunitaulak@gmail.com (S.U.); dhirendrapradhan@gmail.com (D.K.P.); yampokharel@gmail.com (Y.P.P.); 5 Kerala Forest Research Institute, Thrissur District, Thrissur 680653, India; tvsajeev@gmail.com; 6 Food and Agriculture Organization of the United Nations, 00153 Rome, Italy; Shiroma.Sathyapala@fao.org; 7 Food and Agriculture Organization of the United Nations Regional Office for Asia and the Pacific, Bangkok 00153, Thailand; Illias.Animon@fao.org

**Keywords:** Himalaya, invasive species, Nepalese ants, new record, species richness

## Abstract

**Simple Summary:**

There is little information available about the species diversity and distribution patterns of Nepalese ants, as well as their importance in maintaining forest health. A survey of forest ants was conducted in Nepal to learn about their diversity, distribution, and role in forest management. Ants were collected using vegetation beating, sweeping, and hand collection methods in eastern, central, and western Nepal. Seventy ant species from thirty-six genera and six subfamilies were recorded in the study. The research also discovered five genera and nine species new for the country, as well as eight tramp species, four of which are major ecological, agricultural, and/or household pests. The study discovered that western Nepal and the Siwalik region have a relatively high ant diversity. Ant diversity was found to decrease with increasing elevation. The assessment of ant diversity using multiple sampling methods that cover all seasons and forest types may be useful in obtaining complete ant diversity data. Early intervention through sustainable forest management initiatives would aid in preventing invasive ant incursions in the forests of Nepal.

**Abstract:**

The information available on the diversity of ant species and their distribution and interaction with forest health in Nepal remains limited. As part of a nationwide project on forest health, we conducted inventories to assess the diversity and distribution of forest ants and their role in forest management in Nepal. Ants were collected from 187 plots of 10 m × 10 m size along the north–south belt transects in eastern, central, and western Nepal. We used vegetation beating, sweeping, and hand collection methods in selected forest types. In each transect, we designed six plots in each major forest type (Sal, *Schima–Castanopsis*, and broadleaf mixed forests) and three plots each in deodar, *Alnus*, riverine, and *Cryptomeria* forests. We recorded 70 ant species from 36 genera and six subfamilies. This includes five genera and nine species new for the country, as well as eight tramp species, four of which are major ecological, agricultural, and/or household pests. Our study indicates that forest ant species richness is high in western Nepal and the Siwaliks, and it decreases as elevation increases. The high diversity of ant species in the forests of Nepal needs to be assessed with further exploration using multiple sampling methods covering all seasons and forest types. Ants can be useful indicators for ecosystem management and human impacts on forests. Reports of invasive ants in Nepalese forests indicate the relevance of urgent interventions through sustainable forest management initiatives to prevent future incursions.

## 1. Introduction

Ants (family: Formicidae) occur in almost all terrestrial ecosystems, while tropical regions harbor peaks of their diversity [1]. Some ants are key predators [2,3], ecosystem engineers [4,5,6], seed dispersers [7,8], and biocontrol agents [9,10]. However, some ant species are notorious pests of households, agriculture, and forests. Five ant species (*Anoplolepis gracilipes*, *Linepithema humile*, *Pheidole megacephala*, *Wasmannia auropunctata*, and *Solenopsis invicta*) are highly damaging invaders and listed amongst the 100 worst invasive alien species of the world [11]. Ant diversity is noted to be higher in undisturbed primary forests than in secondary forests [12] and is often considered as an indicator of biodiversity and forest health [13,14]. There are two major ant species distribution patterns: decreasing with increasing elevations [15] and mid-elevation peaks [16].

Nepal has been home to eight subfamilies, 48 genera, and 133 species of ants [17,18]. Considering the unique geographical and ecological diversity, many ant species are yet to be discovered, and their ecology (diversity and distribution) is yet to be studied in the country. Since the first description of two ant species from Nepal, *Aphaenogaster pachei* and *Myrmica pachei*, by Forel [19], Nepalese ants have received occasional attention from scientists. Nepalese myrmecology is still in its early stages. There have previously been no publications based on a focused systematic survey of Nepalese ants. Collingwood [20] published the first Nepalese ant list, which included 34 species, while Subedi et al. [17] published the recent updated checklist, which included 128 species. Twenty-one ant species have Nepal as type locality, and nine of them are endemic to the country [17]. The majority of the current information about Nepalese ants is based on hand-collected specimens. This implies the need to conduct systematic surveys using multiple collection methods. However, a dearth of taxonomic expertise and equipped infrastructure in the country pose a challenge in the study of ants.

Forest biodiversity conservation is an important objective of forest management [21,22,23]. Old-growth forests support greater biodiversity than plantation forests [24]. Plantation forests can also play a role in conserving biodiversity [25]. An assessment of forest biodiversity is essential to ensure ecosystem integrity and the sustainability of ecosystem functions [26]. Inventories of entire forest biota are almost impossible, and, thus, bioindicators have been used in monitoring. Ants are ideal candidates in monitoring ecosystem conditions because they occur everywhere and are numerically abundant in both intact and disturbed habitats [27,28], can be easily sampled [29], and are sensitive and rapid responders to environmental variables [27,30]. Ants have been widely used as bioindicators for ecosystem management and biodiversity restoration [31], and, further, they are a useful tool in predicting human impacts on forests [32]. Detecting the presence or absence of invasive or native ‘weedy’ ants can provide valuable information for monitoring [31]. It is extremely difficult to eradicate invasive ants once they have been introduced into natural ecosystems, where they can have a variety of direct and indirect effects on native ant and non-ant taxa [33]. As a result, it is critical to plan ahead of time and take the necessary precautions to avoid possible incursions.

With ants being indicators of forest health, the interaction of ants and forest health must be studied closely; the assessment of the association of ants with different forest regimes is imperative. The present study focuses on the inventory of ants in various forest types along the longitudinal (east, central, and west) and latitudinal (north to south in Tarai, Siwalik, middle mountains, and high mountains) gradients. It aims to provide a baseline for further research on forest–ant interaction and sustainable forest management in Nepal.

## 2. Materials and Methods

### 2.1. Field Sampling

**Figure 1:**
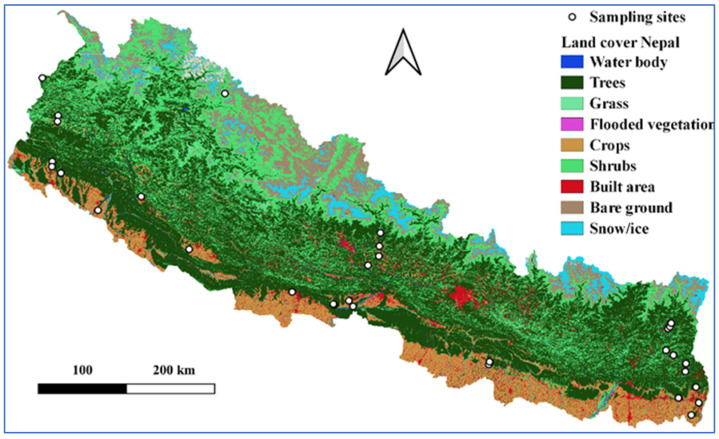
Land cover map of Nepal showing sampling sites.

Ants were sampled from 27 September to 22 October 2020 along south to north belt transects in the eastern, central, and western regions of Nepal. The study area covered the selected forests in Tarai and Siwalik and the mid- and high-mountain regions of 14 districts (Table 1 and Figure 1). The sampling was carried out using sweeping, beating in low vegetation, and hand collection methods. In each belt transect, we selected representative forest types for the sampling sites. In each forest type, we laid two 100 m parallel transects at least 250 m away from each other; six sampling plots (10 m × 10 m) in the major forest types, such as Sal, *Schima–Castanopsis*, and broadleaf mixed forests; and three plots in deodar, *Alnus*, riverine, and *Cryptomeria* forests. Altogether, 187 plots were sampled, including those of the eastern (66), central (66), and western (55) regions. The sampling locations are plotted in the land cover map of Nepal available at arcgis.com [34] using QGIS 3.16 [35]. The following site abbreviations are used in the paper:

WT: western Tarai, WS: western Siwalik, WMM: western mid-mountain, CT: central Tarai, CS: central Siwalik, CMM: central mid-mountain, ET: eastern Tarai, ES: eastern Siwalik, EMM: eastern mid-mountain, EHM: eastern high mountain.

### 2.2. Specimen Processing and Identification 

The specimens were preserved in 95% alcohol and were first sorted by morphospecies. At least one specimen of each morphospecies from a plot was point mounted. The morphological examination of the point-mounted specimens was carried out with a Coslab MSZ-115 stereo zoom microscope. The samples were deposited at the entomology lab, Forest Research Training Centre (FRTC), Ministry of Forests and Environment and included copies of each species in the Central Department Zoology Museum of Tribhuvan University (CDZMTU). Standard taxonomic keys (such as [36,37,38]) were used for subfamily and generic level identification. Species level identification was based on available identification keys (such as [18,39,40,41,42,43,44,45,46,47,48,49,50,51,52]). Species characteristics were compared with original descriptions, and specimens were also tallied with type images available at AntWeb (https://www.antweb.org accessed on 1 October 2021) and AntWiki (https://www.antwiki.org accessed on 1 October 2021). 

### 2.3. Data Analysis 

Species richness of ants was computed at plot, transect, district, and forest type levels. The distribution frequency of a genus was recorded, and an accumulation curve was developed in order to test whether the laid plots were sufficient to inventory the ants. We prepared curves for incidence data considering presence–absence data of ant species at western, central, and eastern regional level. The sampling effort was further evaluated using a jackknife first-order richness estimator 100 permutation species–accumulation curve [53]. The cumulative number of species versus sample plots in the eastern, western, and central regions was used to create a species–accumulation curve. The distribution of ant genera was analyzed at regional level to be precise to Tarai and Siwalik and the hills/mountains of the eastern, western, and central regions of Nepal and presented in a chord diagram. The distribution of ants and altitude was regressed to see the association of ants along the elevational gradient. R program [54] was used to plot the diagrams. Non-metric multidimensional scaling (NMDS) was performed using the PAST software package [55] to visualize differences in distribution of ant genera among the western, central, and eastern regions based on the Bray–Curtis similarity index.

## 3. Results

### 3.1. Ant Species Diversity with New Records for Nepal

More than 1150 specimens were collected during the survey, and we identified 70 ant species or morphospecies representing 36 genera and 6 subfamilies (Dolichoderinae, Ectatomminae, Formicinae, Myrmicinae, Ponerinae, and Pseudomyrmecinae) from 368 occurrences in eastern, central, and western Nepal (Supplementary Materials S1 and S2). Myrmicinae was the most diverse subfamily with 13 genera and 30 species, followed by Formicinae (11 genera and 24 species), Ponerinae (6 genera and 7 species), Dolichoderinae (4 genera and 6 species), Pseudomyrmecinae (1 genus and 2 species), and Ectatomminae (1 genus and 1 species), in the study area. *Crematogaster* and *Camponotus* were the most abundant genera in terms of individuals collected and occurrences, respectively (Table 2). They were also recorded as the most speciose genera, with six species each. Of all the species, *Oecophylla smaragdina* was observed most often, with 37 recorded independent occurrences. The genus *Pheidole* was recorded in 9 transects with 32 independent occurrences. *Crematogaster* (42 occurrences), *Camponotus* (40), and *Nylanderia* (38) were recorded in eight transects each (Table 2). Other commonly occurring genera with over ten occurrences include *Lophomyrmex* (31), *Technomyrmex* (20), *Polyrhachis* (10), *Lepisiota* (10), *Tetramorium* (10), *Myrmicaria* (10), and *Meranoplus* (10) (Supplementary Materials S2). Nine of the thirty-six genera were observed only once during the study (Supplementary Materials S2). 

**Figure 2:**
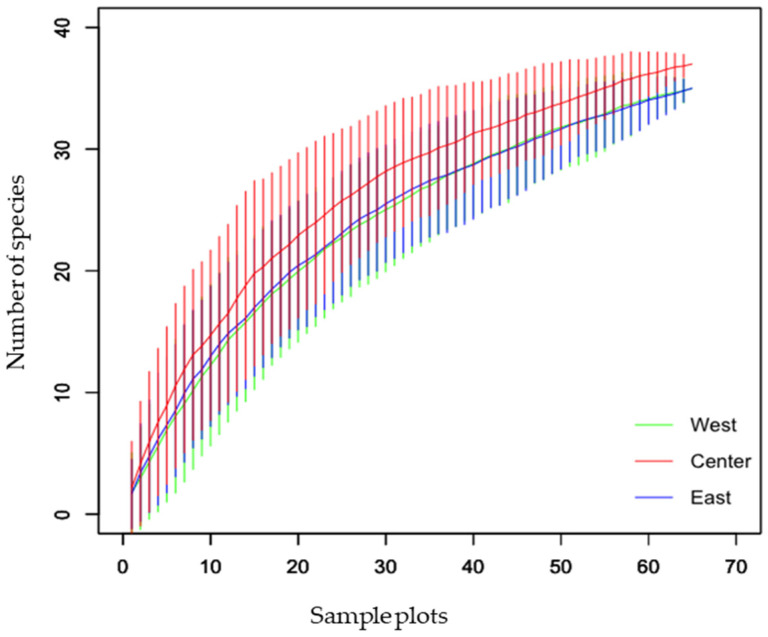
Sample-based species–accumulation curve of ant species in western, central, and eastern regions of Nepal.

Five genera, namely *Ochetellus*, *Tapinoma*, *Colobopsis*, *Pseudolasius*, and *Plagiolepis*, are new generic records for the country. With the identification of 33 valid species from 70 collected species, a total of 9 species (*Ochetellus glaber*, *Tapinoma melanocephalum*, *Gnamptogenys bicolor*, *Colobopsis vitrea*, *Polyrhachis laevissima*, *Polyrhachis punctillata*, *Aphaenogaster beesoni*, *Carebara affinis*, and *Diacamma scalpratum*) represent new records for Nepal. It is worth mentioning that some of our morphospecies are likely new to science, such as *Temnothorax* sp. 1 and 2, which are most closely related to *T. simlensis* but different from it and each other, and sp. 3, which is most closely related to *T. inermis* but different from it, in order to claim them as new species. These species will be described in the near future. The sample-based accumulation curve for the species recorded from three regions, namely eastern, central, and western regions, indicated that the sample plots taken were sufficient to record most of the ant species, as the curves show a trend of plateauing (Figure 2); however, additional sampling is required to inventory the total species richness in the study area.

### 3.2. Tramp and Invasive Ant Species

We found tramp/invasive ants in eleven of the twenty-one forest types studied, indicating that Nepalese forests are vulnerable to tramp/invasive species encroachment (Table 3). We identified eight widespread tramp species, namely *Brachyponera chinensis*, *Cardiocondyla wroughtoni*, *Monomorium pharaonis*, *Ochetellus glaber*, *Paratrechina longicornis*, *Tapinoma melanocephalum*, *Tetramorium lanuginosum*, and *Trichomyrmex destructor*, across the sampling sites. Two of these species, *O. glaber* and *T. melanocephalum*, were discovered in Nepal for the first time. The tramp/invasive species were recorded in both plantations and natural forests. No specific trend was observed regarding the distribution of these tramp ants depending on forest type; however, the majority of them were found in the Sal forest. There were no tramp/invasive species found in the high mountains. Amongst these tramp/invasive species, *M. pharaonis*, *P. longicornis*, *T. melanocephalum*, and *T. destructor* are major pests. These pest species were recorded in tropical forests at elevations lower than 1000 m. However, the impact of these pest species on Nepalese forests has yet to be quantified. 

### 3.3. Distribution of Ant Species

**Figure 3:**
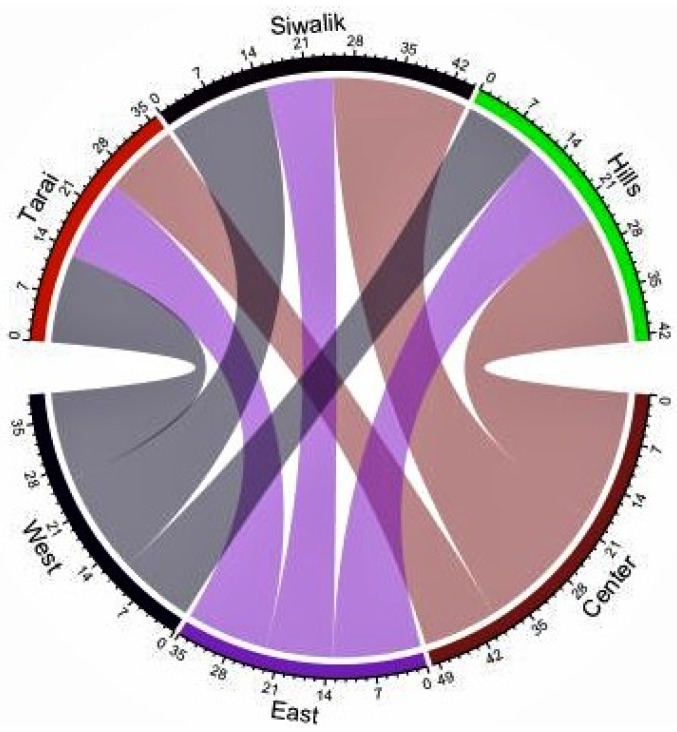
Chord diagram showing shared ant genera in Tarai and Siwalik and the hills of western, central, and eastern regions of Nepal.

The distribution of ant genera in the forests of Nepal has been evaluated as per the physiographic zones, forest types, and elevation gradients. The western region had a slightly higher number of genera (26), followed by the central (23) and eastern (22) regions, with 27 genera recorded in Siwalik (WS-15, CS-20, ES-10) followed by 26 genera in the hills (WMM-11, CMM-19, EMM-13, EHM-3) and 18 in Tarai (WT-13, CT-11, ET-12) (Figure 3). The most diverse forest was Sal forest (29 genera), followed by the mixed broadleaf forest (12), *Dalbergia sissoo–Acacia catechu* forest (11), managed Sal forest (11), champ forest (10), and pine forest (10) (see Supplementary Materials S3). 

**Figure 4:**
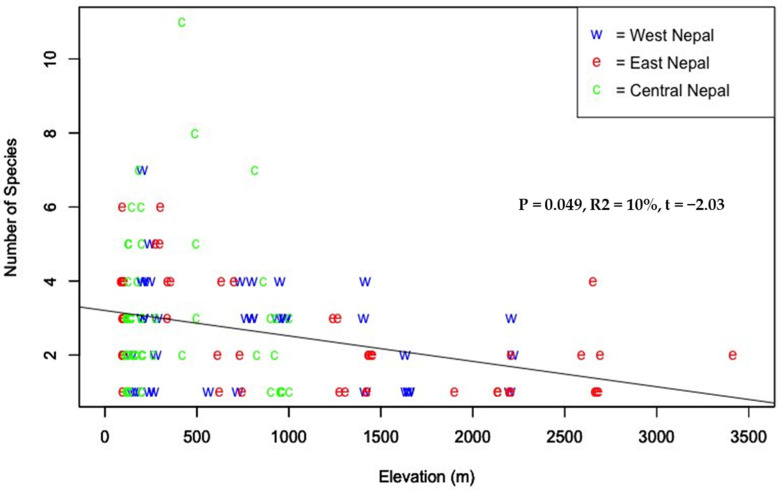
Species richness of ants along elevation gradient in Nepal.

Elevation ranges are provided for all of the genera identified in this study (see Supplementary Materials S2). Ant species richness showed a decreasing pattern with the increase in elevation (Figure 4). The majority of the ant species were recorded at elevations below 1000 m (Figure 4). We recorded *Nylanderia*, *Lepisiota*, *Crematogaster*, *Myrmicaria*, *Pheidole*, and *Brachyponera* at a wide elevation range, while *Lasius*, *Aphaenogaster*, *Myrmica*, and *Temnothorax* were recorded at higher altitudes (above 2000 m) only (Supplementary Materials S2). We recorded *Gnamptogenys*, *Colobopsis*, *Trichomyrmex*, *Diacamma*, *Ectomomyrmex*, *Leptogenys*, and *Pseudoneoponera* from a narrow range in lower altitudes (Supplementary Materials S2). 

**Figure 5:**
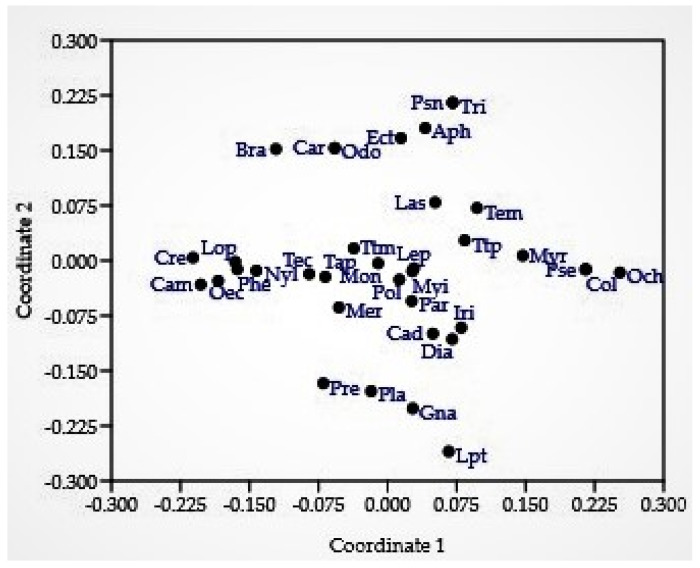
Non-metric dimensional scaling (Bray–Curtis similarity index). *Legend genera*: Aph: *Aphaenogaster*, Bra: *Brachyponera*, Cam: *Camponotus*, Cad: *Cardiocondyla*, Car: *Carebara*, Col: *Colobopsis*, Cre: *Crematogaster*, Dia: *Diacamma*, Ect: *Ectomomyrmex*, Gna: *Gnamptogenys*, Iri: *Iridomyrmex*, Las: *Lasius*, Lep: *Lepisiota*, Lpt: *Leptogenys*, Lop: *Lophomyrmex*, Mer: *Meranoplus*, Mon: *Monomorium*, Myr: *Myrmica*, Myi: *Myrmicaria*, Nyl: *Nylanderia*, Och: *Ochetellus*, Odo: *Odontoponera*, Oec: *Oecophylla*, Par: *Paratrechina*, Phe: *Pheidole*, Pla: *Plagiolepis*, Pol: *Polyrhachis*, Pre: *Prenolepis*, Pse: *Pseudolasius*, Psn: *Pseudoneoponera*, Tap: *Tapinoma*, Tec: *Technomyrmex*, Tem: *Temnothorax*, Ttm: *Tetramorium*, Ttp: *Tetraponera*, Tri: *Trichomyrmex*.

The distribution and association of ant genera revealed that the ants from genera *Leptogenys* dissimilar to that of *Pseudoneoponera* and *Trichomyrmex* and that of *Ochetellus* are distinct from *Crematogaster* and *Camponotus*. The habitats of the ants associated with genera *Monomorium*, *Tetramorium*, *Polyrhachis*, and *Lepisiota* are close to each other (Figure 5).

## 4. Discussion

### 4.1. Ant Species Diversity

This study represents the first nationwide survey of forest ants in Nepal. Species lists are important tools for species conservation because they provide a solid understanding of the current state of the biota, which can serve as a foundation for conservation actions [56]. The species–accumulation curve revealed that the curve approached the asymptote but was not completely leveled off (Figure 2). Despite the fact that the majority of the species were captured in the study areas, additional sampling efforts involving multiple collection methods would result in the capture of a few more species. *Crematogaster* (208 individuals, 42 occurrences, 6 species) and *Camponotus* (181 individuals, 40 occurrences, 6 species) were the most diverse genera. Of all the species, *Oecophylla smaragdina* was recorded most often (38 occurrences) during the study, while the genus *Pheidole* was recorded from Tarai and Siwalik and the hills of the western, central, and eastern regions. Seventeen genera were represented by only one species in our collection, and eight genera were collected from a single site. In Nepal, the richness of Oriental genera (such as *Oecophylla*, *Polyrhachis*, *Prenolepis*, *Carebara*, *Lophomyrmex*, *Leptogenys*, *Meranoplus*, and *Tetraponera*) outnumbered the Palaearctic genera (such as *Myrmica*, *Lasius*, and *Temnothorax*) as in southern China [57]. Several genera were found to be common throughout Tarai and Siwalik and the hills of western, central, and eastern regions, while others were found to be peculiar to a specific location (Figure 3 and Figure 5), with the Palearctic genera being reported at high elevations. Although it is difficult to make an exact comparison of this study with other studies because objectives, sampling methods, the area covered, and identification levels vary, our findings are consistent with ant inventories in our neighboring countries, particularly in dominant subfamilies and commonly occurring genera, such as southern China [57], Yunnan, China [58], northwestern Siwalik, India [59], southwest China [60], Jammu and Kashmir, India [61]. Furthermore, our findings agree with those of a previous study that recognized *Camponotus*, *Pheidole*, and *Crematogaster* as the three most species-rich genera on a global scale [62]. Weaver ants (*O. smaragdina*) are conspicuous arboreal ants with more than 2700 site records from 21 countries [10]. They share beneficial traits with other ant species and cause cascading effects to lower trophic levels, reducing pest number and damage in orchards and forests [63]. 

### 4.2. Distribution of Ants in Nepal 

The variation in the distribution of the forest ants of Nepal with elevation is in line with other studies. The highest number of ant genera in our study was recorded below 1000 m asl, and there was a decreasing pattern with the increase in elevation (Figure 4) as reported by Subedi and Budha [64] when they extrapolated available elevation-related records of ants of Nepal from 200 m to 4550 m. A decreasing pattern is one of the most common patterns of ant species richness [64,65]. A number of studies from different parts of the world also reported a decreasing pattern of species richness with elevation, such as those from Mount Kinabalu [66], the Mediterranean and the oro-Mediterranean parts of Montenegro [67], rainforest in subtropical Queensland [68], wet forest on Costa Rica’s Atlantic slope [69], Imbak Canyon [70], Hengduan Mountains [71], and eastern Himalaya [15]. The most common pattern of species richness along the elevation gradient in the Nepal Himalaya seems to be a unimodal pattern [72,73,74]. However, the elevation with maximum richness is not similar for different taxa [75,76]. 

### 4.3. Ants and Forest Health

In Nepal, we recorded eight common tramp species, including *M. pharaonis*, *P. longicornis*, *T. melanocephalum*, and *T. destructor*. These four species are amongst twelve cosmopolitan ants that have become significant ecological, agricultural, and/or household pests [77]. Wetterer [78] considered *P. longicornis* to be the most widely dispersed ant species, with records from both the Old and New Worlds in both the northern and southern hemispheres. Tramp and invasive species are geographically widespread, and observations of functional groups and biogeography may help to better understand the factors that contribute to their spread [79]. Most of the widespread ants belong to the subfamily Myrmicinae and, more specifically, to the following functional groups: cryptic, opportunist, and generalized myrmicine [79]. 

Ants are indispensable components for the maintenance and appropriate functioning of most terrestrial ecosystems and resulting ecosystem services and disservices [80]. Ant species composition strongly influences seed dispersal by ants [81], and ant communities are very useful for the rapid assessment of terrestrial ecosystem health [82]. Ants play a crucial ecological role by helping in soil formation and by increasing its fertility [83,84]. Three ant species, including *O. smaragdina*, have been identified as predators of the sal heartwood borer (*Hoplocerambyx spinicornis*) in nature [85]. In our study, we also frequently observed *O. smaragdina* in Sal forests.

Of the 42 cosmopolitan ants, only 12 species are major ecological, agricultural, and/or household pests [77], and 4 of them were recorded in Tarai and Siwalik and the mid-mountains of the western, central, and eastern regions of the country. Invasive ant characteristics, such as greater abundance, aggressive behavior, and attraction to high-carbohydrate resources, may result in a high and low risk of deleterious effects on plants, resulting in a negative conservation impact [86]. Bharti and Sharma [16] revealed that the most invasive ant species occurred at lower altitudes, indicating their correlation with the disturbance of the ecosystem.

When used as bioindicators, ants can help detect early signs of habitat disturbance and can help develop management strategies [87]. Invasive ant eradication is critical for biodiversity conservation; however, more than half of the global eradication efforts were not successful [88]. The initiatives aimed to eradicate 11 species around the world, namely *Anoplolepis gracilipes*, *Linepithema humile*, *Pheidole megacephala*, *Wasmannia auropunctata*, *Solenopsis invicta*, *S. geminata*, *T. melanocephalum*, *Lepisiota frauenfeldi*, *Myrmecia brevinoda*, *Monomorium indicum*, and *P. longicornis* [88]. Two of the eleven species causing problems around the world, namely *P. longicornis*, and *T. melanocephalum*, were also recorded in our study. Other invasive ants, such as *A. gracilipes* and *P. megacephala* that are common in neighboring countries are not yet known in Nepal. We believe that studies focusing on the tramp and invasive species of ants in urban areas should be conducted in order to complete their inventory in the country. 

MoFE [89] identified invasive species expansion as one of the direct drivers of deforestation and forest degradation, posing a very high effect in Tarai and the Siwaliks, a low effect in the mid-mountains, and a very low effect in the high mountains. Using ants as bioindicators to assess ecosystem health, Bharti and Sharma [16] discovered that the invasive species are seriously threatening the native species of the primary and secondary forests of the Himalaya. They also stated that while ant invasions are currently limited to lower mountain ranges, as global temperatures rise, they may spread to highlands. 

The vast majority of insect species are herbivores, but only nine insect orders, including Hymenoptera (ants, wasps, bees, hornets, and sawflies), have species that feed on live plants [90]. Larger carpenter ants (*Camponotus* sp.) may excavate solid wood in living tree trunks, making them major forest pests [91]. In Nepal, pests and pathogens have invaded all types of forests, resulting in serious consequences, such as forest degradation and biodiversity loss [92].

The early detection of invasive species, pests, and pathogens is critical for preventing damage because early control activities are more feasible and effective [92,93]. Thorough monitoring of invasive ant species encroachment in forest areas is critical for protecting fragile ecosystems and maintaining sound forest health. With increased global travel and trade, several other undocumented tramp/invasive ants may occur or be introduced into the country, particularly in urban areas. A more concentrated collection of tramp/invasive ants in urban areas is necessary to identify them and prevent their possible incursion into natural forests.

## 5. Conclusions

Our study represents the most extensive ant diversity survey in Nepal that has been published to date. The study recorded 70 ant species from 36 genera and 6 subfamilies, revealing slightly higher richness in western Nepal and in the Siwaliks. The study also reported five genera and nine species that are new to Nepalese ant fauna. Ant species richness declined with elevation. The study further concluded that the invasive species have already been introduced in Nepalese forests, including four serious pests. Based on this small-scale survey, we conclude that Nepalese forests feature a high species diversity and richness for ants. However, the forest biodiversity is compounded by invasive ants. Ant species can also be a useful indicator for the management of forest ecosystems and human impacts. Our results demonstrate the relevance of urgent interventions through extensive research and sustainable forest management initiatives to prevent future incursions. 

## Figures and Tables

**Table 1:** Site characteristics.

Sites	Sampling Plots	District/s	Forest Types	Latitude (N)	Longitude (E)	Altitude Range (m)
WT	13	Kailali	Sal, riverine forests	28.48313–28.81801	80.67581–81.12818	143–208
WS	18	Kailali, Surkhet, Dang	*Eucalyptus* plantation, broadleaf mixed, Sal, riverine forests	28.09533–28.92537	80.56907–82.20147	172–243
WMM	24	Darchula, Dadeldhura	Pine, *Alnus*, *Quercus*, deodar forests	29.345–29.76724	80.40099–82.54359	722–2239
CT	15	Sarlahi, Rupandehi	*Eucalyptus* plantation, teak plantation, Sal forest	26.99305–27.69817	83.4002–85.69093	105–167
CS	27	Nawalpur	Sal, *Dalbergia sissoo–Acacia catechu*, mixed broadleaf riverine forests	27.55949–27.62469	83.87765–84.10534	108–204
CMM	24	Tanahun, Lamjung	Champ plantation, Sal, *Acacia*, *Schima–Castanopsis*, *Bombax ceiba*, *Alnus nepalensis* forests	27.59218–28.33335	84.16049–84.40139	262–1011
ET	18	Jhapa	Sal, teak, mixed broadleaf forests	26.4746–26.70515	87.84115–88.07256	87–135
ES	6	Ilam	Sal forest	26.76225–26.76538	88.03876–88.04182	276–357
EMM	24	Ilam, Panchthar	Sal, *Schima–Castanopsis*, pine, Uttis, *Cryptomeria* forests	26.92539–27.18556	87.69814–87.93221	613–2208
EHM	18	Taplejung	Laurel, mixed, oak, *Abies* forests	27.36369–27.42682	87.72441–87.76557	2569–3645

**Table 2:** Top seven ant genera by abundance of individuals and occurrences in eastern, central, and western regions of Nepal.

Ant Genera	Individuals Collected			Occurrences		
	Eastern	Central	Western	Eastern	Central	Western
*Crematogaster*	95	36	77	16	19	7
*Camponotus*	11	130	40	28	5	7
*Oecophylla*	14	91	35	19	9	9
*Lophomyrmex*	34	24	60	8	15	8
*Pheidole*	22	64	30	13	8	11
*Nylanderia*	21	39	28	13	10	15
*Technomyrmex*	23	18	7	7	11	2

**Table 3:** Tramp/invasive ants occurring in different forest types and regions of Nepal.

Tramp/Invasive Ant Species	Forest Types	Regions
*Brachyponera chinensis*	*Alnus* forest, mixed broadleaf forest, *Quercus* forest, Sal forest	WT, WS, WMM
*Cardiocondyla wroughtoni*	Champ plantation, Sal forest	CMM
*Monomorium pharaonis*	Champ plantation, *Dalbergia sissoo–Acacia catechu* forest, *Eucalyptus camaldulensis* plantation, Sal forest	WT, WS, CMM, ES
*Ochetellus glaber*	Pine forest	EMM
*Paratrechina longicornis*	Sal forest	WT, CS, CMM
*Tapinoma melanocephalum*	Champ plantation, riverine forest, Sal forest, Managed Sal forest, *Schima–Castanopsis* forest	WS, WT, CS, CMM
*Tetramorium lanuginosum*	Sal forest, *Schima-Castanopsis* forest	ES, EMM
*Trichomyrmex destructor*	Riverine forest, Sal forest	WT, WS

## Data Availability

All the associated data are available in the manuscript and Supplementary Materials.

## Supplementary Materials

The following are available online at https://www.mdpi.com/article/10.3390/insects12121128/s1, Supplementary Material S1: Species list of the ants from the study sites; Supplementary Material S2: Recorded ant genera, their elevational distribution and sites of occurrence; Supplementary Material S3: Occurrence of ant genera in different forest types.
