# Supplementary material for: Diversity and Distribution of Forest Ants (Hymenoptera: Formicidae) in Nepal: Implications for Sustainable Forest Management
Source: Insects. 2021 Dec 17;12(12):1128. doi: 10.3390/insects12121128 (PMC8707472; doi:10.3390/insects12121128)
Supplement: Supplementary file 1 [file insects-12-01128-s001.zip › insects-1434441-supplementary.pdf]

Supplementary Material S1. Species list of the ants from the study sites

Dolichoderinae (4 genera, 6 species)

Iridomyrmex anceps (Roger, 1863)

Iridomyrmex sp 1

Ochetellus glaber (Mayr, 1862)

Tapinoma melanocephalum (Fabricius, 1793)

Technomyrmex sp 1

Technomyrmex sp 2

Ectatominae (1 genus, 1 species)

Gnamptogenys bicolor (Emery, 1889)

Formicinae (11 genera, 24 species)

Camponotus (Tanaemyrmex) sp 1

Camponotus (Tanaemyrmex) sp 2

Camponotus (Tanaemyrmex) sp 3

Camponotus mutilarius Emery, 1893

Camponotus rufoglaucus (Jerdon, 1851)

Camponotus sericeus (Fabricius, 1798)

Colobopsis vitrea (Smith, 1860)

Lasius magnus Seifert, 1992

Lepisiota sp 1

Lepisiota sp 2

Oecophylla smaragdina (Fabricius, 1775)

Pseudolasius sp 1

Nylanderia sp 1

Nylanderia sp 2

Nylanderia cf. *smythiesii*

Paratrechina longicornis (Latreille, 1802)

Plagiolepis sp 1

Plagiolepis sp 2

Polyrhachis lacteipennis Smith, 1858

Polyrhachis laevissima Smith, 1858

Polyrhachis punctillata Roger, 1863

Prenolepis fustinoda Williams & LaPolla 2016

Prenolepis naoroji Forel, 1902

Myrmicinae (13 genera, 30 species)

Aphaenogaster beesoni Donisthorpe, 1933

Cardiocondyla wroughtonii (Forel, 1890)

Cardiocondyla sp 2

Carebara affinis (Jerdon, 1851)

Myrmicinae

Crematogaster sp 1

Crematogaster sp 2

Crematogaster sp 3

Crematogaster sp 4

Crematogaster sp 5

Crematogaster sp 6

Lophomyrmex ambiguus Rigato, 1994

Lophomyrmex cf. *bedoti*

Meranoplus bicolor (Guérin-Méneville, 1844)

Monomorium cf. *sahlbergi*

Monomorium pharaonis (Linnaeus, 1758)

Myrmica weberi Elmes & Radchenko, 2009

Myrmica indica Weber, 1950

Myrmicaria brunnea gr sp 1

Pheidole sp 1

Pheidole sp 2

Pheidole sp 3

Pheidole sp 4

Pheidole sp 5

Temnothorax sp 1

Temnothorax sp 2

Temnothorax sp 3

Tetramorium lanuginosum Mayr, 1870

Tetramorium cf. *obesum*

Tetramorium sp 1

Trichomyrmex destructor (Jerdon, 1851)

Ponerinae (6 genera, 7 species)

Brachyponera chinensis (Emery, 1895)

Diacamma scalpratum (Smith, 1858)

Diacamma sp 1

Ectomomyrmex sp 1

Leptogenys diminuta (Smith, 1857)

Odontoponera denticulata (Smith, 1858)

Pseudoneoponera bispinosa (Smith, 1858)

Pseudomyrmicinae (1 genus, 2 species)

Tetraponera rufonigra (Jerdon, 1851)

Tetraponera allaborans (Walker, 1859)

Supplementary Material S2. Recorded ant genera, their elevational distribution and sites of occurrence

Ant genera	Number of species	Elevation		Total number of occurrences	Sites of occurrence	Districts
		Min.	Max			
Iridomyrmex	2	418	1455	4	CMM, EMM	TN, IL, PN
Ochetellus	1	1455	1455	1	EMM	PN
Tapinoma	1	183	937	8	WS, CS, CMM	KL, NP, TN, LM
Technomyrmex	2	93	1265	20	WT, CS, CMM, ET, ES, EMM	KL, NP, TN, LM, JP, IL, PN
Gnamptogenys	1	144	183	2	CS	NP
Camponotus	6	87	1407	40	WT, WS, WMM, CT, CS, CMM, ET, EMM	KL, DA, DR, SA, RP, NP, TN, LM, JP, PN
Colobopsis	1	338	338	1	ES	IL
Lasius	1	2199	2210	2	EMM, WMM	DD, PN
Lepisiota	2	144	2208	10	WMM, CS, CMM, EMM	DR, DD, NP, TN, PN
Oecophylla	1	87	983	37	WT, WS, CT, CS, CMM, ET, ES	KL, SU, RP, NP, TN, LM, JP, IL
Pseudolasius	1	276	276	1	ES	IL
Nylanderia	3	87	2135	38	WT, WS, WMM, CT, CMM, ET, ES, EMM	KL, DR, DD, SA, RP, NP, TN, LM, JP, IL, PN
Paratrechina	1	175	418	3	WT, CS, CMM	KL, NP, TN
Plagiolepis	2	119	859	3	CS, CMM	NP, TN
Polyrhachis	3	94	898	10	WT, CT, CS, CMM, ET, EMM	KL, SA, NP, TN, JP, PN
Prenolepis	3	119	487	7	CT, CS, CMM	SA, NP, TN
Aphaenogaster	1	2218	2218	1	WMM	DD
Cardiocondyla	2	128	1416	5	WMM, CS, CMM	DR, NP, TN
Carebara	1	802	802	1	WS	SU
Crematogaster	6	93	2208	42	WT, WS, CT, CS, CMM, ET, ES, EMM	KL, DR, RP, NP, TN, LM, JP, IL, PN
Lophomyrmex	2	93	2218	31	WS, WMM, CS, CMM, ET, ES, EMM	KL, DR, DD, NP, TN, LM, JP, IL, PN
Meranoplus	1	94	1000	10	WT, WS, CT, CS, CMM, ET	KL, SA, NP, TN, JP, PN
Monomorium	2	115	952	7	WT, WS, CT, CS, CMM, ES	KL, SA, NP, TN, IL
Myrmica	2	2590	2693	5	EHM	TP
Myrmicaria	1	95	2664	10	WS, CT, CS, ET, EHM	KL, RP, NP, JP, TP
Pheidole	5	95	2208	32	WT, WS, WMM, CT, CS, CMM, ET, ES, EMM	KL, DR, RP, NP, TN, LM, JP, IL, PN
Temnothorax	3	2210	3413	3	WMM, EHM	DD, TP
Tetramorium	3	128	1275	10	WT, CS, CMM, ES, EMM	KL, NP, LM, IL, TP
Trichomyrmex	1	204	245	3	WT, WS	KL
Brachyponera	1	284	2210	7	WS, WMM	KL, SU, DD
Diacamma	2	98	144	4	CT, CS, ET	SA, NP, JP
Ectomomyrmex	1	265	265	1	WS	KL

Leptogenys	1	198	198	1	CS	NP
Odontoponera	1	734	1416	3	WMM	DR
Pseudoneoponera	1	204	204	1	WT	KL
Tetraponera	2	98	983	4	WS, CMM, ET, EMM	KL, TN, JP, PN

WT: Western Terai, WS: Western Siwalik, WMM: Western Mid Mountain, CT: Central Terai, CS: Central Siwalik, CMM: Central Mid Mountain, ET: Eastern Terai, ES: Eastern Siwalik, EMM: Eastern Mid Mountain, EHM: Eastern High Mountain

DA: Dang, DR: Darchula, DD: Dadeldhura, IL: Ilam, JP: Jhapa, KL: Kailali, LM: Lamjung, NP: Nawalpur, PN: Panchthar, RP: Rupandehi, SA: Sarlahi, SU: Surkhet, TP: Taplejung, TN: Tanahun

Supplementary Material S3. Occurrence of ant genera in different forest types

**Forest types**	**Number of genera**	**Ant genera**
Acacia forest	4	*Lophomyrmex, Nylanderia, Crematogaster, Prenolepis*
Alnus forest	6	*Camponotus, Tetramorium, Lophomyrmex, Pheidole, Nylanderia, Brachyponera*
Apis forest	1	*Temnothorax*
Bombax ceiba forest	2	*Technomyrmex, Camponotus*
Champ plantation	10	*Polyrhachis, Crematogaster, Monomorium, Oecophylla, Tapinoma, Nylanderia, Cardiocondyla, Camponotus, Plagiolepis, Lophomyrmex*
Cryptomeria forest	2	*Crematogaster, Nylanderia*
Dalbergia sissoo-Acacia catechu forest	11	*Camponotus, Meranoplus, Myrmicaria, Tetramorium, Cardiocondyla, Crematogaster, Monomorium, Technomyrmex, Prenolepis, Pheidole, Lophomyrmex*
Deodar forest (Plantation)	1	*Nylanderia*
Eucalyptus camaldulensis plantation	6	*Prenolepis, Meranoplus, Camponotus, Monomorium, Polyrhachis, Diacamma*
Laurel forest	3	*Myrmica, Temnothorax, Myrmicaria*
Mixed broadleaf forest	12	*Lophomyrmex, Nylanderia, Crematogaster, Pheidole, Oecophylla, Diacamma, Tetraponera, Myrmicaria, Technomyrmex, Camponotus, Brachyponera, Ectomomyrmex*
Mixed broadleaf riverine forest	6	*Meranoplus, Camponotus, Pheidole, Plagiolepis, Prenolepis, Technomyrmex*
Mixed forest	1	*Myrmica*
Pine forest	10	*Odontoponera, Pheidole, Lepisiota, Cardiocondyla, Camponotus, Lophomyrmex, Lepisiota, Crematogaster, Iridomyrmex, Ochetellus*
Quercus forest	6	*Temnothorax, Lasius, Brachyponera, Lepisiota, Aphaenogaster, Lophomyrmex*
Riverine forest	7	*Trichomyrmex, Tapinoma, Oecophylla, Pheidole, Meranoplus, Camponotus, Polyrhachis*
Sal (*Shorea robusta*) forest	29	*Pheidole, Trichomyrmex, Crematogaster, Nylanderia, Paratrechina, Oecophylla, Technomyrmex, Tetramorium, Monomorium, Polyrhachis, Pseudoneoponera, Meranoplus, Camponotus, Tetraponera, Lophomyrmex, Tapinoma, Carebara, Brachyponera, Odontoponera, Lepisiota, Myrmicaria, Plagiolepis, Gnamptogenys, Diacamma, Iridomyrmex, Cardiocondyla, Prenolepis, Pseudolasius, Colobopsis*

Sal forest (Managed)	11	*Polyrhachis, Leptogenys, Myrmicaria, Camponotus, Nylanderia, Oecophylla, Prenolepis, Crematogaster, Tapinoma, Pheidole, Meranoplus*
Schima-Castanopsis forest	9	*Lophomyrmex, Technomyrmex, Meranoplus, Pheidole, Oecophylla, Crematogaster, Tapinoma, Tetramorium, Iridomyrmex*
Teak Plantation	2	*Camponotus, Nylanderia*
Utis forest	4	*Crematogaster, Lepisiota, Lasius, Pheidole*
